# External temperature and distance from nearest entrance influence microclimates of cave and culvert‐roosting tri‐colored bats (*Perimyotis subflavus*)

**DOI:** 10.1002/ece3.5841

**Published:** 2019-12-11

**Authors:** Samantha J. Leivers, Melissa B. Meierhofer, Brian L. Pierce, Jonah W. Evans, Michael L. Morrison

**Affiliations:** ^1^ Natural Resources Institute Texas A&M University College Station Texas; ^2^ Department of Wildlife and Fisheries Sciences Texas A&M University College Station Texas; ^3^ Wildlife Diversity Program Texas Parks and Wildlife Department Boerne Texas

**Keywords:** actual water vapor pressure, cave, culvert, hibernation, microclimate, *Perimyotis subflavus*, temperature, tri‐colored bat

## Abstract

Many North American bat species hibernate in both natural and artificial roosts. Although hibernacula can have high internal climate stability, they still retain spatial variability in their thermal regimes, resulting in various “microclimates” throughout the roost that differ in their characteristics (e.g., temperature and air moisture). These microclimate components can be influenced by factors such as the number of entrances, the depth of the roost, and distance to the nearest entrance of the roost. Tri‐colored bats are commonly found roosting in caves in winter, but they can also be found roosting in large numbers in culverts, providing the unique opportunity to investigate factors influencing microclimates of bats in both natural and artificial roost sites. As tri‐colored bats are currently under consideration for federal listing, information of this type could be useful in aiding in the conservation and management of this species through a better understanding of what factors affect the microclimate near roosting bats. We collected data on microclimate temperature and microclimate actual water vapor pressure (AWVP) from a total of 760 overwintering tri‐colored bats at 18 caves and 44 culverts. Using linear mixed models analysis, we found that variation in bat microclimate temperatures was best explained by external temperature and distance from nearest entrance in both caves and culverts. External temperature had a greater influence on microclimate temperatures in culverts than caves. We found that variation in microclimate AWVP was best explained by external temperature, distance from nearest entrance, and proportion from entrance (proportion of the total length of the roost from the nearest entrance) in culvert‐roosting bats. Variation in microclimate AWVP was best explained by external temperature and proportion from entrance in cave‐roosting bats. Our results suggest that bat microclimate temperature and AWVP are influenced by similar factors in both artificial and natural roosts, although the relative contribution of these factors differs between roost types.

## Graphical abstract

Tri‐colored bats are commonly found winter roosting in caves, but they can also be found roosting in culverts, providing the unique opportunity to investigate factors influencing bat microclimate temperatures in both natural and artificial roost sites. We collected data on microclimate temperatures and actual water vapor pressure near roosting bats in 18 caves and 44 culverts. Our results suggest that bat microclimate components are influenced by the same factors in both artificial and natural roosts, although the relative contribution of these factors differs between roost types.

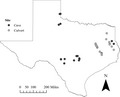

## 1 INTRODUCTION

The majority of temperate bat species in North America hibernate during winter months. Of those species that hibernate, both natural and artificial subterranean structures are used as hibernacula. Caves are often used by bats and provide an environment of stable internal temperatures and high, or stable, humidity regimes (Kuenzi, Downard, & Morrison, 1999; Perry, 2013; Speakman & Thomas, 2003) that allow bats to enter a state of torpor in which body temperature and energy expenditure are greatly reduced (Geiser, 2004). Broadly, internal cave climate measures can be affected by environmental characteristics—such as airflow and external temperature—and physical characteristics of the cave—such as placement on the landscape, diameter, shape, and depth (Perry, 2013). Additional factors which add complexity in understanding internal cave climates include size and position of entrance(s), air circulation, and water infiltration (Tuttle & Stevenson, 1978).

Although caves can have high internal climate stability, they still retain spatial variability in their thermal regimes (Perry, 2013), resulting in various “microclimates” throughout the cave that differ in their characteristics (e.g., temperature and air moisture). Bats will selectively choose microclimates in which to roost based on individual variation in factors such as sex, body condition, rates of evaporative water loss (EWL), and heat loss (Boyles, Dunbar, Storm, & Brack, 2007; Jonasson & Willis, 2011; Reeder & Moore, 2013). For example, little brown bats (*Myotis lucifugus*) with low energy reserves (i.e., low body fat) choose cold microclimate temperatures to enter a deeper state of torpor and thus avoid the energetic costs of arousal from hibernation (Boyles et al., 2007). Conversely, little brown bats with larger energy reserves choose warm microclimate temperatures to minimize costs associated with hibernation (e.g., decreased predator avoidance and sleep deprivation).

Tri‐colored bats (*Perimyotis subflavus*) are a species of North American bat commonly found overwintering in caves and other subterranean locations. Tri‐colored bats are widely distributed in the eastern half of North America and were once considered common, but populations have declined substantially due to their susceptibility to white‐nose syndrome (WNS; Bernard & McCracken, 2017; Frick et al., 2017; Ingersoll, Sewall, & Amelon, 2013). The species is currently under review for listing under the Endangered Species Act by the United States Fish and Wildlife Service (USFWS, 2017). Tri‐colored bats are distributed throughout Texas and are unique in that they can be found roosting, not just in caves, but also in culverts (Demere et al., 2017; Meierhofer & Demere, 2017; Meierhofer et al., 2019; Sandel et al., 2001; Walker, Sandel, Honeycutt, & Adams, 1996). Culverts assist with drainage by allowing water to flow under roads. They vary in shape and size and are constructed from materials such as metal and concrete. Within Texas, an estimated 70% of the state's tri‐colored bat population hibernates in culverts during the winter months (J. Evans, per. comm.), suggesting that culverts provide a suitable range of microclimates from which bat can choose to roost. Although artificial structures have been recognized as winter roosts for bats for many years (e.g., Mirowsky, Horner, Maxey, & Smith, 2004; Sandel et al., 2001; Walker et al., 1996), the ways in which they are comparable to natural roosts remain relatively unknown. As tri‐colored bats hibernate in both caves and culverts, we are provided with the unique opportunity to investigate factors (structural and environmental) influencing bat microclimate (i.e., air temperature and air moisture near bats) in both natural and artificial hibernacula structures. Should bat microclimate factors in both roost types (i.e., caves and culverts) be influenced by the same environmental and structural factors (e.g., external temperature, and distance from an entrance), we may be provided with information regarding at least one way in which culverts are providing suitable environments from which hibernating bats are able to select. Alternatively, should microclimate factors be influenced by different environmental and structural factors between roost types, we can determine roost type‐specific factors that contribute to providing suitable environments from which bats can select. As tri‐colored bats are currently under consideration for federal listing, a better understanding of how internal microclimate is impacted by environmental and structural factors of caves and culverts will be necessary for tailoring site‐specific conservation and management action plans for this species in Texas and other states.

## 2 METHODS

**Figure 1:**
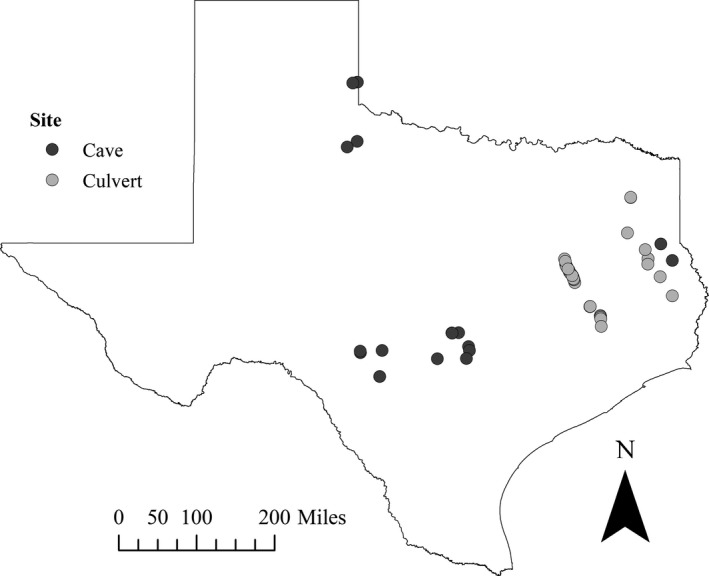
Sixty‐two tri‐colored bat winter roosts (18 caves and 44 culverts) surveyed across Texas from December 2016 to February 2017, December 2017 to March 2018, and November 2018 to January 2019. Data collected included external temperature, height of bat, total length of site, distance from nearest entrance, and relative humidity (from which to calculate actual water vapor pressure)

We surveyed caves and culverts for overwintering tri‐colored bats across the state of Texas. In total, we surveyed 62 tri‐colored bat winter roosts (18 caves and 44 culverts) during daylight hours from December 2016 to February 2017, December 2017 to March 2018, and November 2018 to January 2019 (Figure 1). We surveyed caves and culverts between one and three times between 2016 and 2019 (no more than one survey per winter, per site). Due to limitations in accessing caves on private lands, caves were surveyed opportunistically. We obtained locations of caves with assistance from the Texas Speleological Society (TSS), Texas Cave Management Association (TCMA), Texas Grottos, Texas Parks and Wildlife biologists, and private landowners. Permission to access caves was obtained from the relevant entities (e.g., landowner). We obtained information on locations of historic culvert bat colonies from previous literature (Sandel et al., 2001; Walker et al., 1996), from the Texas Department of Transportation (TxDOT), and from biologists. In addition to historic colonies, we randomly selected 77 10 × 10 km grid cells across the state using the Generalized Random Tessellation Stratified (GRTS) design (Stevens & Olsen, 2004) of the North American Bat Monitoring Program (NABat) in order to sample additional culverts. Within each grid cell, we identified and surveyed all potential box culverts using Google Earth Pro 7.3.2 (Google, 2018) with the aid of culvert location data provided by the TxDOT. We considered culverts potential sampling sites if they were large enough to enter and if they were located in a place that could be safely accessed (e.g., there was a safe place in which to pull over when driving).

Due to the large number of bats found in some winter roosts, we selected a sample of tri‐colored bats within sites for data collection so as to work within time constraints and reduce disturbance. To the best of our ability, we staggered our sampling efforts to collect data from bats distributed throughout each site. In some instances where we were unable to reliably record data from bats (e.g., when bats were out of reach, not in a safely accessible area), we recorded data from the next bat encountered. We recorded two microclimate factors for each bat: air temperature (°C) as a measure of microclimate temperature and relative humidity (%) as a measure of microclimate air moisture. We took these recordings within 5 cm of each bat using a temperature and humidity pen (Extech 445580) which was held in place until values ceased to fluctuate. This pen allows for a simultaneous display of temperature and relative humidity and has built‐in self‐calibration to ensure accurate recordings. At the time of each survey, we recorded external air temperature (°C) using the temperature and humidity pen. For each bat, we recorded the height of bat (m; distance from bat to ground) and recorded total length of site (m) using a laser distance measurer (Tuirel T100) or using cave maps. As some caves had multiple entrances, we measured the distance from each bat to the nearest visible or known entrance (as determined by cave maps) using the laser distance measurer. All methods followed ASM guidelines (Sikes & The Animal Care and Use Committee of the American Society of Mammalogists, 2016) and were approved by the Texas A&M Institutional Animal Care and Use Committee (IACUC 2015‐0296).

### 2.1 Data analysis

To take into consideration the potential influence of social thermoregulation on microclimate factors (Kerth, 2008; Russo et al., 2017), we removed data taken from bats that were roosting in a cluster (i.e., one bat touching at least one other bat). This resulted in a total of 95 temperature and humidity recordings from bats roosting within 18 caves. We collected 665 microclimate temperature recordings from bats roosting within 44 culverts but, due to missed data collection, we collected 653 microclimate humidity recordings.

As relative humidity can be an inappropriate metric to use to describe the dryness of the air when temperature varies, we calculated microclimate actual water vapor pressure (AWVP) in order to create a measure of microclimate air moisture (Anderson, 1936; Kurta, 2014; Kurta & Smith, 2014). We converted microclimate relative humidity to microclimate AWVP by first calculating the saturation water vapor pressure (SWVP) for every microclimate temperature recorded and then multiplying SWVP by microclimate relative humidity (Kurta & Smith, 2014).

We used R v. 3.4.1 (R Core Team, 2018) to perform all statistical analyses. We used linear mixed‐effect models (LMEs) to determine factors that influence both microclimate temperature and AWVP. In all models, we included site ID as a random factor and entered combinations of the following fixed factors into the LME models: external temperature, height of bat, total length of site, and distance from nearest entrance. To take into consideration the potential interaction of total length of site and the distance from nearest entrance on bat microclimate factors (Elliot & Clawson, 2001; Smithson, 1991), we also calculated an additional variable to enter into the models called “proportion from entrance.” For caves, we calculated proportion from entrance by dividing the bat's distance from its nearest entrance (as some caves had multiple entrances) by the total length of the cave. This resulted in a value between 0 and 1 that indicated the location of the bat within the cave, so that a bat with a value closer to 1 was located further from a cave entrance and a bat with a value closer to 0 indicated a bat located closer to an entrance of the cave. As all surveyed culverts had two entrances, the point furthest from the entrance was the center of the culvert. Therefore, to calculate proportion from entrance for culverts, we divided the bat's distance from nearest entrance by the total length/2; a bat with a value closer to 1 was located closer to the center of the culvert whereas a bat with a value closer to 0 was at located to an entrance of the culvert. We standardized all fixed factors prior to analysis and checked for multicollinearity using variance inflation factors (VIFs). VIFs were <3 in all instances, suggesting that no or very minimal multicollinearity between fixed factors existed.

We analyzed data separately for caves and culverts for each bat microclimate factor (i.e., temperature and AWVP). Using the lmer function in the lme4 package (Bates, Mächler, Bolker, & Walker, 2015), we created 31 LMEs (including the null model) using all combinations of the fixed factors to investigate microclimate temperature in caves, and 31 LMEs using the same method to investigate microclimate temperature in culverts. This method was repeated to investigate microclimate AWVP near bats in caves and culverts. We ranked models using AICc (Akaike's information criterion with correction for small sample sizes) in order to determine models of best fit. Models were considered candidate models if they had an AICc weight ≥10% of the AICc weight of the top model. We determined the amount of variation explained by each model by calculating both the marginal *R* ^2^ (variance explained by fixed effects only) and conditional *R* ^2^ (variance explained by both fixed and random effects; Nakagawa & Schielzeth, 2013) using the r‐squared.GLMM function in the MuMIn package. Where LMEs resulted in multiple candidate models, we used a model averaging approach to incorporate model uncertainty and produced model averaged estimates (Burnham & Anderson, 1998, 2002). However, due to the potential pitfalls of using model averaging for ecological data (Banner & Higgs, 2016; Cade, 2015; Harrison et al., 2018), we also present model averaged predictions using candidate models as an additional approach to interpreting the strength and direction of fixed factors (Cade, 2015).

We also conducted simple Welch's *t* tests on unstandardized data (to account for unequal sample sizes, Fields, Jeremy, & Fields, 2012) to examine the difference in microclimate temperature and microclimate AWVP between cave and culvert‐roosting bats and to describe differences in fixed factors between caves and culverts. We considered *p*s ≤ .05 significant.

## 3 RESULTS

We found no statistically significant difference in microclimate temperature between caves and culverts (Table 1). However, there was a statistically significant difference in microclimate AWVP between caves and culverts, with bats in caves roosting in higher AWVP than culvert‐roosting bats. Caves were statistically significantly longer than culverts and showed greater variability in length. There was a statistically significant difference in distance from nearest entrance: bats roosting in caves were closer to a roost entrance than bats roosting in culverts. Similarly, there was a statistically significant difference in proportion from entrance: cave‐roosting bats were closer to a roost entrance than culvert‐roosting bats, which were more likely to be roosting closer toward the middle of the culvert (i.e., further from an entrance). There was a statistically significant difference in external temperature between cave and culvert‐roosting bats, with higher external temperatures recorded for culvert‐roosting bats. There was no significant difference in height of bat between caves and culverts.

**Table 1:** Means and standard deviations of dependant variables and fixed factors for tri‐colored bats roosting in caves and culverts in Texas in winter with associated Welch's *t* tests

Factors	Culverts		Caves		*df*	*T*	*p*
	$\bar{X}$	*SD*	$\bar{X}$	*SD*			
Microclimate temperature (°C)	15.95	3.71	15.80	3.19	133.17	−0.44	.66
Microclimate actual water vapor pressure (kHa)	0.12	0.05	0.13	0.04	134.51	3.09	<.01
Total length of site (m)	124.98	43.19	305.00	764.13	94.09	2.30	.02
External temperature (°C)	17.04	4.29	16.10	4.72	117.34	1.85	.07
Height of bat (m)	1.64	0.34	1.61	0.65	101.26	−0.42	.67
Distance from nearest entrance (m)	25.75	18.40	15.22	12.00	165.39	−7.41	<.01
Proportion from entrance	0.44	0.28	0.28	0.24	134.18	−5.99	<.01

Note

Proportion from entrance describes bat location within a roost (0 = at an entrance, 1 = furthest point from an entrance).

**Figure 2:**
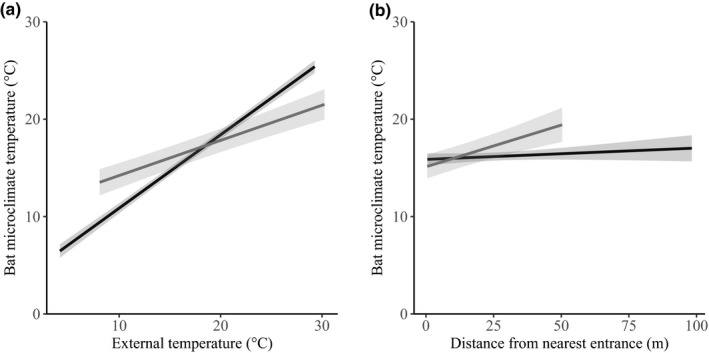
Model averaged predictions and 95% confidence intervals (shaded areas) of the effects of (a) external temperature and (b) distance from nearest entrance on tri‐colored bat microclimate temperature. The black line represents culvert‐roosting bats, and the gray line represents cave‐roosting bats. The range of data is limited by the minimum and maximum value for each *x* variable for each roost type

We categorized 10 LME models as candidate models for explaining variance in microclimate temperature for culvert data (Table 2). The top model included external temperature (*β* = 3.24, *SE* = 0.09) and distance from nearest entrance (*β* = 0.21, *SE* = 0.07). Model averaging suggested that external temperature was the only significant predictor of microclimate temperature of culvert‐roosting bats, but distance from nearest entrance approached significance (Table 4). Model averaged predictions (Figure 2) suggest that external temperature has the greatest influence on bat microclimate temperature than other variables.

**Table 2:** Linear mixed‐effect candidate models, null models, and worst models explaining variation in tri‐colored bat microclimate temperature in both culverts and caves

	Models	AICc	AICc weight	ΔAICc	Marginal *R* ^2^	Conditional *R* ^2^
Culverts	**External temperature + Distance from nearest entrance**	**2,672.34**	**0.29**	**0**	**.71**	**.81**
	**External temperature + Distance from nearest entrance + Total length**	**2,673.65**	**0.15**	**1.31**	**.71**	**.81**
	**External temperature + Distance from nearest entrance + Height of bat**	**2,674.05**	**0.12**	**1.72**	**.71**	**.81**
	**External temperature + Distance from nearest entrance + Total length + Proportion from entrance**	**2,674.48**	**0.10**	**2.14**	**.71**	**.81**
	**External temperature + Proportion from entrance**	**2,674.61**	**0.09**	**2.27**	**.71**	**.81**
	**External temperature + Distance from nearest entrance + Total length + Height of bat**	**2,675.53**	**0.06**	**3.19**	**.71**	**.81**
	**External temperature + Distance from nearest entrance + Height of bat + Proportion from center**	**2,675.85**	**0.05**	**3.51**	**.71**	**.81**
	**External temperature + Distance from nearest entrance + Total length + Height of bat + Proportion from entrance**	**2,676.32**	**0.04**	**3.99**	**.71**	**.81**
	**External temperature + Height of bat + Proportion from entrance**	**2,676.55**	**0.03**	**4.21**	**.71**	**.81**
	**External temperature + Total length + Proportion from entrance**	**2,676.62**	**0.03**	**4.28**	**.71**	**.81**
	*Null (intercept only)*	3,372.02	0	699.68	—	.47
	Distance from nearest entrance + Height of bat + Proportion from entrance*	3,376.55	0	704.22	<.01	.47
Caves	**External temperature** + **Distance from nearest entrance**	**395.26**	**0.35**	**0**	**.37**	**.83**
	**External temperature + Distance from nearest entrance + Total length**	**395.87**	**0.25**	**0.61**	**.42**	**.83**
	**External temperature + Distance from nearest entrance + Height of bat**	**397.08**	**0.14**	**1.82**	**.37**	**.83**
	**External temperature + Distance from nearest entrance + Total length + Height of bat**	**397.76**	**0.10**	**2.50**	**.43**	**.83**
	**External temperature + Distance from nearest entrance + Total length + Proportion from entrance**	**398.08**	**0.08**	**2.82**	**.42**	**.83**
	**External temperature + Distance from nearest entrance + Height of bat + Proportion from entrance**	**399.41**	**0.04**	**4.15**	**.37**	**.83**
	*Null (intercept only)*	471.73	0	76.48	—	.40
	Height of bat*	473.38	0	78.13	<.01	.39

Note

We ranked models according to AICc and they are presented with AICc weights, ΔAICc, and marginal and conditional *R* ^2^. Candidate models are in bold (models were candidate models if they had an AICc weight ≥10% of the AICc weight of the top model), null models are italicized and the worst model is marked with *. We entered the following fixed factors into models: total length of site (m), external temperature (°C), height of bat (distance from bat roosting location to ground; m), distance from nearest entrance (m), and proportion from entrance (0 = at entrance, 1 = furthest point from entrance).

We categorized six LME models as candidate models for explaining microclimate temperature for cave‐roosting bats (Table 3). The top model included external temperature (*β* = 1.70, *SE* = 0.18) and distance from nearest entrance (*β* = 1.04, *SE* = 0.19). Model averaging suggested that distance from nearest entrance and external temperature were the only significant predictors of microclimate temperature for cave‐roosting bats (Table 4). Model averaged predictions (Figure 2) suggested that distance from nearest entrance and external temperature had the greatest influence on bat microclimate temperature.

**Table 3:** Linear mixed‐effect candidate models, null models and worst models explaining variation in tri‐colored bat actual water vapor pressure (AWVP) in both culverts and caves

	Models	AICc	AICc weight	ΔAICc	Marginal *R* ^2^	Conditional *R* ^2^
Culverts	**External temperature + Distance from nearest entrance + Height of bat + Proportion from entrance**	**−2,914.63**	**0.57**	**0**	**.44**	**.79**
	**External temperature + Distance from nearest entrance + Total length + Height of bat + Proportion from entrance**	**−2,913.39**	**0.31**	**1.25**	**.43**	**.78**
	**External temperature + Distance from nearest entrance + Total length + Proportion from entrance**	**−2,911.45**	**0.12**	**3.19**	**.44**	**.78**
	*Null (intercept only)*	−2,516.78	0	397.86	—	.66
	Total length	−2,514.80	0	399.83	<.01	.66
Caves	**External temperature _+_ Proportion from entrance**	**−424.35**	**0.31**	**0**	**.26**	**.88**
	**External temperature + Proportion from entrance + Total length**	**−424.20**	**0.29**	**0.15**	**.24**	**.89**
	**External temperature + Proportion from entrance + Height of bat**	**−422.55**	**0.13**	**1.80**	**.26**	**.88**
	**External temperature + Proportion from entrance + Total length + Height of bat**	**−422.40**	**0.12**	**1.95**	**.26**	**.88**
	**External temperature + Distance from nearest entrance + Total length + Proportion from entrance**	**−421.90**	**0.09**	**2.45**	**.26**	**.88**
	**External temperature + Distance from nearest entrance + Proportion from entrance + Height of bat**	**−420.22**	**0.04**	**4.13**	**.24**	**.89**
	**External temperature + Distance from nearest entrance + Total length + Height of bat + Proportion from entrance**	**−420.08**	**0.04**	**4.27**	**.26**	**.88**
	*Null (intercept only)*	−361.59	0	62.76	—	.42
	Height of bat + Total length*	−357.94	0	66.41	.03	.42

Note

We ranked models according to AICc and they are presented with AICc weights, ΔAICc, and marginal and conditional *R* ^2^. Candidate models are in bold (models were candidate models if they had an AICc weight ≥10% of the AICc weight of the top model), null models are italicized and the worst model is marked with *. We entered the following fixed factors into models: total length of site (m), external temperature (°C), height of bat (distance from bat roosting location to ground; m), distance from nearest entrance (m), and proportion from entrance (0 = at entrance, 1 = furthest point from entrance).

**Table 4:** Model averaged estimates with 95% confidence intervals (CIs) for variables retained in the candidate model sets that predicted tri‐colored bat microclimate temperature and microclimate actual water vapor pressure in both culverts and caves

	Fixed factor	Model averaged *β*	*SE*	95% CI	
				Lower	Upper
Microclimate temperature					
Culverts	**External temperature**	**3.23**	**0.09**	**3.06**	**3.41**
	Distance from nearest entrance	0.26	0.15	−0.03	0.55
	Total length	−0.20	0.23	−0.65	0.24
	Height of bat	0.07	0.14	−0.20	0.34
	Proportion from entrance	−0.02	0.25	−0.51	0.46
Caves	**External temperature**	**1.70**	**0.18**	**1.34**	**2.06**
	**Distance from nearest entrance**	**1.03**	**0.21**	**0.62**	**1.43**
	Total length	0.84	0.63	−0.39	2.07
	Height of bat	−0.13	0.19	−0.51	0.25
	Proportion from entrance	0.07	0.37	−0.64	0.79
Microclimate actual water vapor pressure					
Culverts	**External temperature**	**0.03**	**0.00**	**0.03**	**0.03**
	**Distance from nearest entrance**	**0.02**	**0.00**	**0.01**	**0.02**
	Total length	0.00	0.00	−0.01	0.01
	Height of bat	0.00	0.00	−0.01	0
	**Proportion from entrance**	**−0.01**	**0.00**	**−0.02**	**−0.01**
Caves	**External temperature**	**0.02**	**0.00**	**0.01**	**0.02**
	Distance from nearest entrance	0.00	0.00	−0.01	0.01
	Total length	0.02	0.01	−0.01	0.04
	Height of bat	0.00	0.00	0.00	0.01
	**Proportion from entrance**	**0.02**	**0.00**	**0.01**	**0.03**

Note

Factors in bold indicate coefficients that do not overlap zero and thus are considered significant predictors.

**Figure 3:**
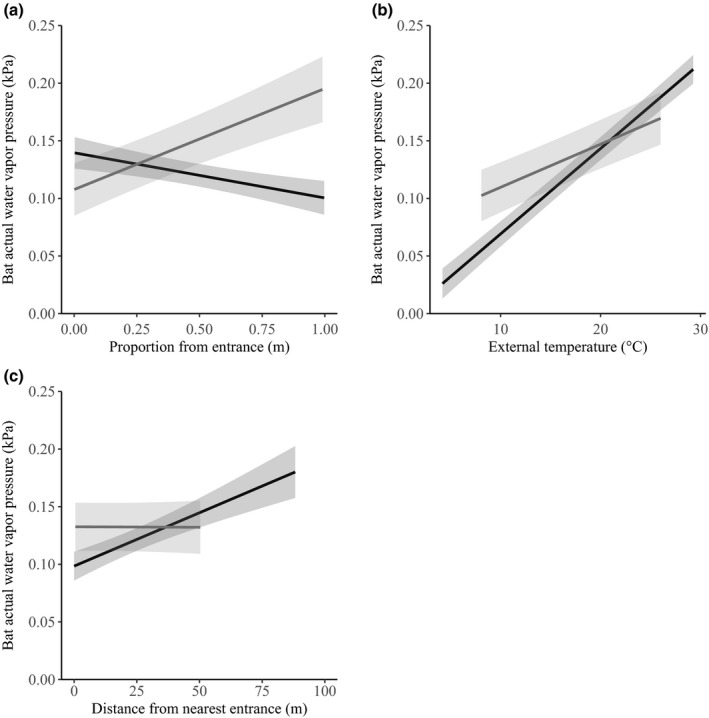
Model averaged predictions and 95% confidence intervals (shaded areas) of the effects of (a) proportion from entrance (0 = at an entrance, 1 = furthest point from an entrance), (b) external temperature, and (c) distance from nearest entrance on tri‐colored bat microclimate actual water vapor pressure (AWVP). The black line represents culvert‐roosting bats, and the gray line represents cave‐roosting bats. The range of data is limited by the minimum and maximum value for each *x* variable for each roost type

We categorized three LME models as candidate models for explaining variance in microclimate AWVP for culvert data (Table 3). The top model included external temperature (*β* = 0.03, *SE <* 0.01), distance from nearest entrance (*β* = 0.02, *SE* < 0.01), height of bat (*β* < −0.01, *SE* < 0.01), and proportion from entrance (*β* < −0.01, *SE* < 0.01). Model averaging suggested that external temperature, distance from nearest entrance, and proportion from entrance were the significant predictors of microclimate AWVP for culvert‐roosting bats (Table 4). Model averaged predictions (Figure 3) suggested that distance from nearest entrance and external temperature had the greatest influence on bat microclimate AWVP.

We categorized seven LME models as candidate models for explaining microclimate AWVP for cave‐roosting bats (Table 2). The top model included external temperature (*β* = 0.02, *SE* < 0.01) and proportion from entrance (*β* = 0.02, *SE* < 0.01). Model averaging suggested that external temperature and proportion from entrance were the only significant predictors of microclimate AWVP for cave‐roosting bats (Table 4). Model averaged predictions (Figure 3) also suggested that proportion from entrance and external temperature had the greatest influence on bat microclimate AWVP.

## 4 DISCUSSION

We found that external temperature and distance from the nearest hibernacula entrance influenced bat microclimate temperature in both caves and culverts. Furthermore, we found that external temperature, distance from the nearest entrance, and proportion from entrance influenced bat microclimate AWVP in both caves and culverts. These factors explained more of the variance in microclimate temperature and AWVP in culvert‐roosting bats than in cave‐roosting bats.

Within caves and culverts, external temperature was the main predictor in explaining bat microclimate temperatures (see Perry, 2013 for a review). Distance from the nearest entrance had a marginally significant influence on microclimate temperatures in culverts and a larger, significant influence on microclimate temperatures in caves. Both cave and culvert‐roosting tri‐colored bats appeared to choose microclimate temperature by selecting roost locations at different distances from the entrances of the hibernacula, with this effect being more pronounced in cave‐roosting bats. Out results support previous research findings that bats which are located closer to an entrance had colder microclimate temperatures than those roosting further away from their nearest entrance (i.e., toward the center of the culvert or the furthest point from an entrance in a cave; Boyles, Boyles, Dunlap, Johnson, & Brack, 2017).

While external temperature and distance from nearest entrance influenced microclimate temperatures within both caves and culverts, coefficient estimates suggested that external temperature had a greater influence on microclimate temperatures in culverts than in caves. Furthermore, the top LME model for culvert data found that distance from entrance and external temperature explained approximately 70% of variance in microclimate temperatures, whereas the top LME model for cave data that also included these factors explained roughly 36% of variance in microclimate temperature. These results suggest that while microclimate temperatures are influenced by external temperatures in both caves and culverts, internal culvert temperatures are more strongly affected by external temperature. This supports datalogger data presented in Meierhofer et al. (2019) that indicates a correlation between external and internal temperatures in both caves and culverts, but with culverts having a stronger correlation with external temperature than caves. Thus, results presented in Meierhofer et al. (2019) suggest greater variability in culvert microclimates than in caves. Culverts may have greater variability in internal temperatures and be more greatly affected by external temperature as a result of their design (generally being straight, with two entrances to allow for air flow) and maintenance (clearing of brush around entrances), whereas not all caves have multiple entrances or may have additional barriers (vegetation) to reduce airflow. This is of importance, as culverts may not always provide microclimates suitable for roosting bats during extreme weather events or as the climate continues to change. Nevertheless, microclimate temperatures recorded by culvert‐roosting bats in this study fell within temperature ranges in which tri‐colored bats have previously been recorded (Webb, Speakman, & Racey, 1995). Tri‐colored bats are considered generalists in terms of their suitable range of temperatures for hibernation, potentially allowing them to utilize culverts as winter roosts where culverts are unsuitable for other species that have stricter temperature requirements (Meierhofer et al., 2019; Webb et al., 1995). Although air temperature may be more variable within culverts than caves, this variability does not preclude tri‐colored bats from using culverts as winter roosts.

Similarly to microclimate temperature, microclimate AWVP was mostly influenced by external temperature in both caves and culverts, with moister air conditions as external temperature increased. Within culverts, AWVP was also influenced by the distance to the nearest entrance whereas proportion from entrance played a greater role in explaining variation in microclimate AWVP in caves. This suggests that microclimate AWVP in culverts is influenced by distance from nearest entrance regardless of the total length of the roost whereas, in caves, the influence of distance from the nearest entrance interacts with the total length of the hibernacula to explain variation in microclimate AWVP. This may well be due to the much larger variation in roost length for caves than culverts: While our largest culvert was approximately 200 m in length, our largest cave was approximately 3,000 m. Nevertheless, both cave and culvert‐roosting bats have the ability to choose their microclimate AVWP based off their intrinsic needs (e.g., rates of evaporative water loss) by alternating their location within a cave or culvert.

Overall, our microclimate temperature models explained approximately 80% of the variance in microclimate temperature data, with our fixed factors explaining approximately 36% and 70% of variance in the data in caves and culverts, respectively. Our microclimate AWVP models explained approximately 78%–89% of the variance in the data, with fixed factors explaining approximately 44% and 26% of variance in the data in caves and culverts, respectively. These results suggest that cave microclimate temperatures have a greater number of factors influencing them than culvert microclimates. Furthermore, the fact that our models for microclimate temperature and AWVP for caves explained approximately 83% and 89% of variance in the data respectively when taking into account both fixed and random effects suggests that there is greater individual variation between caves than culverts. Culverts are essentially simple, straight structures with two entrances, differing mostly by length, width and height. Conversely, caves can have multiple entrances and greater variation in length, depth, complexity, and variation in airflow (see Perry, 2013 for review). Continued data collection at caves would allow further analysis to determine other factors that influence microclimate temperatures by providing more data and thus allowing more complex statistical modeling.

One limitation with our study is that data collection from bats was not completely random. Due to the height or structure of some caves, data collection of bats was limited to those within our reach. As such, the height at which a bat roosts may explain some variance in microclimate temperature and microclimate AWVP, but the variance in our data was too small to recognize this. Future research should find methods to collect microclimate temperature and AWVP data from these bats without increasing disturbance.

Our research provides information on both caves and culverts serving as roosts for hibernating tri‐colored bats. In both caves and culverts, tri‐colored bats can select suitable microclimate temperatures and AVWP by altering their distance from the entrances of the roost. This allows bats to choose a location with microclimate temperature and AWVP based on their intrinsic needs as weather or body condition changes during a season (Boyles et al., 2007). However, external temperature was the driving predictor of microclimate temperature and AWVP, showing that movements within a roost cannot compensate for roosts in locations that do not meet the species' temperature requirements. Indeed, should climate change over time (Lindsey & Dalhman, 2018), tri‐colored bats may be in the position to compensate for rising temperatures by altering their location within a roost that remains within a suitable temperature profile. This may be of particular importance to culvert‐roosting tri‐colored bats, as external temperature appears to influence internal temperatures more so than in caves (Meierhofer et al., 2019). On a broader scale, our research speaks to the potential ways in which artificial roosts can replicate conditions of natural roosts and thus provide suitable habitats for hibernating bats. Furthermore, determining the differences in how structural and environmental characteristics influence microclimate components between roost types also allows wildlife managers to consider roost‐specific management practices for conservation.

## CONFLICT OF INTEREST

None declared.

## AUTHOR CONTRIBUTIONS

All authors contributed to the conception and design of the work or the collection, analysis, and interpretation of data. All authors assisted in drafting or editing the manuscript.

## Data Availability

All data included in analyses are available through Dryad, https://doi.org/10.5061/dryad.c866t1g36
